# Association between back muscle degeneration and spinal-pelvic parameters in patients with degenerative spinal kyphosis

**DOI:** 10.1186/s12891-019-2837-0

**Published:** 2019-10-20

**Authors:** Weiwei Xia, Han Fu, Zhenqi Zhu, Chenjun Liu, Kaifeng Wang, Shuai Xu, Haiying Liu

**Affiliations:** 1 Department of Spinal Surgery, Peking University People’s Hospital, Beijing, China; 2 Department of Respiratory Medicine, Chinese People’s Liberation Army (PLA) General Hospital, Beijing, China

**Keywords:** Degenerative spinal kyphosis, Lumbar back muscle degeneration, Sagittal imbalance, Digital imaging analysis

## Abstract

**Background:**

The paraspinal and psoas muscles have been considered to be essentially important for stabilizing the spinal column, and the muscle degeneration was found to exist in degenerative spinal kyphosis (DSK) patients. However, it is still not clear the relationship between muscle degeneration and spinal-pelvic alignment. The purpose of this study was to determine the correlations between the individual muscle degeneration at each lumbar spinal level and spinal-pelvic parameters in DSK patients.

**Methods:**

The imaging data of 32 patients with DSK were retrospectively analyzed. The fat infiltration (FI) and relative cross-sectional area of muscle (RCSA) were quantitatively measured for multifidus (MF), erector spinae (ES) and psoas (PS) at each spinal level from L1/2 to L5/S1. The correlations were analyzed between RCSA and the sagittal vertical axis (SVA), thoracic kyphosis (TK), thoracolumbar kyphosis (TLK), lumbar lordosis (LL), sacral slope (SS), pelvic tilt (PT) and pelvic incidence (PI).

**Results:**

The FI of MF and ES at L3/4, L4/5 and L5/S1 were higher than that at L1/2 and L2/3. The FI of PS at L4/5 and L5/S1 were lower than that of L1/2, L2/3 and L3/4. The RCSA of ES and PS from L1/2 to L5/S1 gradually increased, whereas the RCSA of ES from L1/2 to S5/S1 gradually decreased. The RCSA of MF at the L1/2 level was negatively correlated SVA (r = − 0.397,*p* = 0.024); the RCSA at L3/4, L4/5 and L5/S1 levels were negatively correlated with TK (r = − 0.364, *p* = 0.04; r = − 0.38, *p* = 0.032; r = − 0.432, *p* = 0.014); the RCSA at L4/5 level was positively correlated with LL (r = 0.528, *p* = 0.002). The RCSA of ES at L3/4 and L4/5 levels were positively correlated with PI (r = 0.377, *p* = 0.037) and SS (r = 0.420, *p* = 0.019).

**Conclusions:**

FI of MF and ES at lower lumbar level is higher than that at upper level, but FI of PS at upper lumbar level is higher than that at lower level. MF and ES have different roles for maintaining the sagittal spinal-pelvic balance.

## Background

Degenerative spinal kyphosis (DSK) is the structural deformity caused by spinal degeneration, which is mainly manifested by the decrease or loss of normal lordosis angle of the lumbar part of the spine or increased kyphosis of the thoracic or thoracolumbar part on the sagittal plane [1]. It has been suggested that DSK is associated with degenerative changes of the spine, such as disc narrowing, collapsed vertebral bodies, or atrophy of lumbar extensor muscles without iatrogenic injury [2, 3].

Muscular atrophy due to denervation, disuse or other causes can manifest in decreased muscular size, increased infiltration by fat and/or connective tissue [4]. The paraspinal and psoas muscles have been considered to be essentially important for stabilizing the spinal column, and fatty infiltration in muscle decreases the proportion of contractile tissue capable of producing force [5, 6]. A previous study showed that the fat infiltration of paraspinal muscles in patients with degenerative lumbar flat back were higher than healthy subjects using T2 weighted MR Image analysis [7]. However, they only measured the whole back muscle compartment in that study. In addition, the paraspinal muscles show asymmetry in patients with degenerative scoliosis, i.e., the cross-sectional area (CSA) of the multifidus muscle was significantly smaller, and the percentage of fat infiltration of both the multifidus and longissimus muscles was significantly higher on the concave side of the curve at all spinal levels [8]. This indicates that the different extent of back muscles degeneration could be accompanied with different extent of spinal deformity. Therefore, the individual measurements of each muscle, including multifidus, erector spinae and psoas, should have great value for showing their specific degenerative characteristics and roles affecting spinal sagittal alignment in DSK patients.

The spine and pelvic are two important components in maintaining sagittal balance which can be manifested by spinal-pelvic parameters. The balance, however, is often disrupted in DSK patients. It was reported that the extensor muscle volume in the lower lumbar spine is related to the magnitude of the sagittal curvature [9]. However, they only measured the extensor muscles as a whole at only L3/4 spinal level, they did not identify whether the relationship found between muscle volume and curvature was the same in both the multifidus and erector spinae at each lumbar spinal level. Another study showed a moderate correlation between the multifidus CSA and global spine alignment and spinopelvic alignment in degenerative lumbar scoliosis patients [10]. To the best of our knowledge, no studies until now have analyzed the influence of back muscles degeneration at each lumbar spinal level on the spinal-pelvic sagittal balance.

Therefore, we hypothesize that the degeneration of paraspinal muscles may also affect the sagittal spinal-pelvic balance in DSK patients. The aims of the present study were (1) to quantitatively measure the degree of degeneration of multifidus, erector spinae and psoas muscles at each spinal level from L1/2 to L5/S1 in DSK patients by MRI with digital image analysis, and (2) to determine the correlation between individual muscle degeneration at each lumbar spinal level and spinal-pelvic parameters.

## Methods

### Demographic characteristics

The medical records of 32 patients with complete whole spine X-rays and lumbar MRI when attending our hospital from March 2016 to May 2018 were retrospectively analyzed. The DSK patients were diagnosed by 1) characteristic clinical features: a forward stoop with difficulty walking, adaptive postural changes in an attempt to maintain a normal standing position, such as pelvic tilt; and 2) radiographic evaluations using a full-length 36 in. standing lateral radiograph of the entire spine [11]. The inclusion criteria for the study patients were: no history of tumor, tuberculosis, infection, trauma and other definite pathological changes; no history of scoliosis (cobb angle of coronal scoliosis is less than 10°) and spinal surgery. The patients’ weight were assessed based on BMI recommended for Asians by WHO: 18.5~22.9 (normal weight), 23~24.9 (overweight), and BMI ≥ 25 (obese) [3, 12]. This study was conducted according to the principles in the Declaration of Helsinki.

### Muscle quantitative measurements

The MRI image data were acquired on the 1.5 T Sigma whole body imaging system (General Electric, WI). The patients were placed in the supine position, with their legs straight and the lumbar spine in a neutral posture. Measurements were performed from L1/2 to L5/S1, which were obtained parallel to the superior endplate of the lower vertebra at each level; therefore, a total of five slices per patient (160 slices in total) were evaluated [4].

**Fig. 1:**
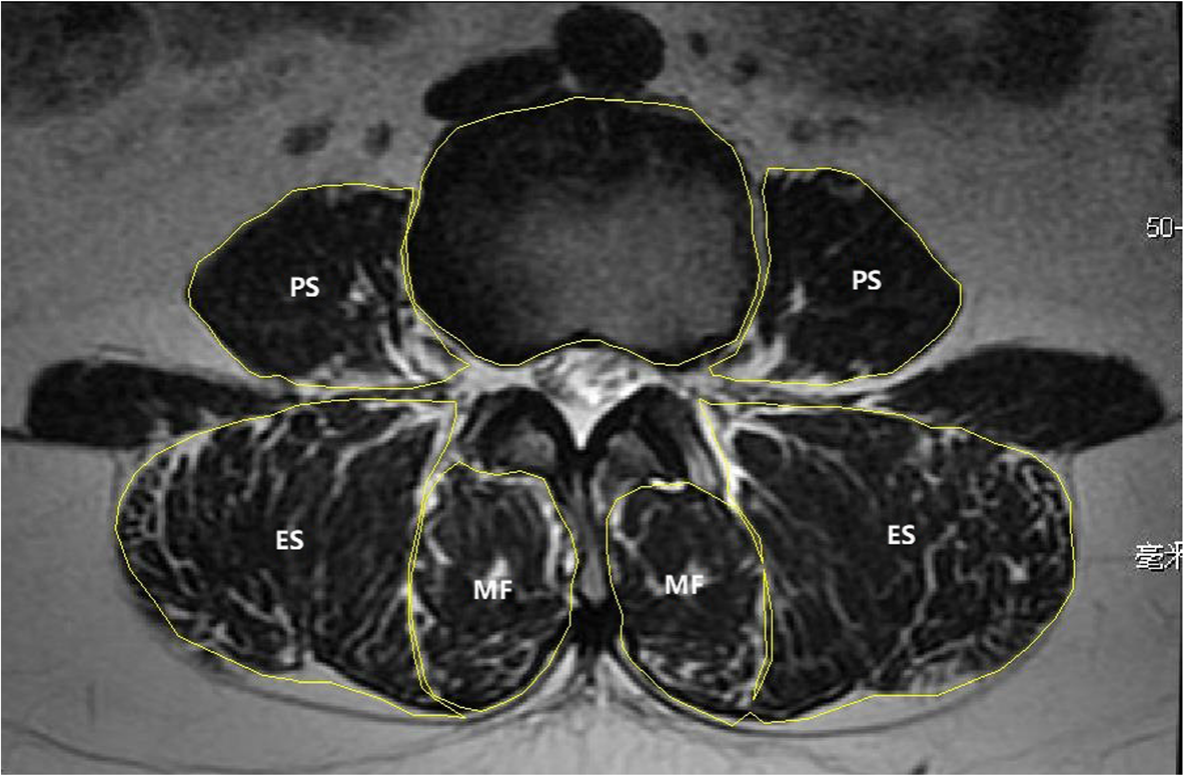
Axial T2-weighted MR image demonstrating measurement of the CSA of different muscle groups and the VB by creating ROIs. PS = psoas muscle, ES = erector spinae, MF = multifidus, VB = vertebrae body

Six regions of interest (ROI) for the muscles were manually defined per slice: the ROI for the multifidus, the erector spinae and the psoas muscle were defined bilaterally [4, 13] (Fig. 1). The muscle measurements performed were total CSA (muscle size) and functional CSA (FCSA, lean muscle). Muscle CSA was determined by constructing the border of each muscle using polygon tool from the ImageJ software (version 1.52, National Institutes of Health, USA). The FCSA was estimated according to the method proposed by Ranson et al. [14], setting the threshold range from 0 to 120 for the grey scale to only include those pixels representing lean muscle content from each muscle CSA. This method has also been used for calculating the fat infiltration (FI), i.e., FI = (CSA-FCSA)/CSA [15]. To compensate for the bias caused by the relative body size of the individual on muscle CSA, we calculated the relative CSA (RCSA), i.e., dividing the muscle FCSA by the CSA of the superior endplate of the lower vertebrae at each spinal level [4]. RCSA was used to evaluate the lumbar muscularity to stabilize the spine column. Each image was assessed two times, and the average value was calculated as the final result. The reliability of the RCSA measure was performed selectively using images at L3/4 spinal level of all included patients. ICC intra-rater was excellent for RCSA for multifidus, erector spinae and psoas [ICC = 0.984 (95% CI = 0.968–0.992); ICC = 0.997 (95% CI = 0.993–0.998); ICC = 0.998 (95% CI = 0.995–0.999)]. High values also were found for the ICC inter-rater RCSA measures to multifidus, erector spinae and psoas [ICC = 0.968 (95% CI = 0.935–0.984); ICC = 0.991 (95% CI = 0.982–0.996); ICC = 0.983 (95% CI = 0.966–0.992)].

### Spinal-pelvic parameters

**Fig. 2:**
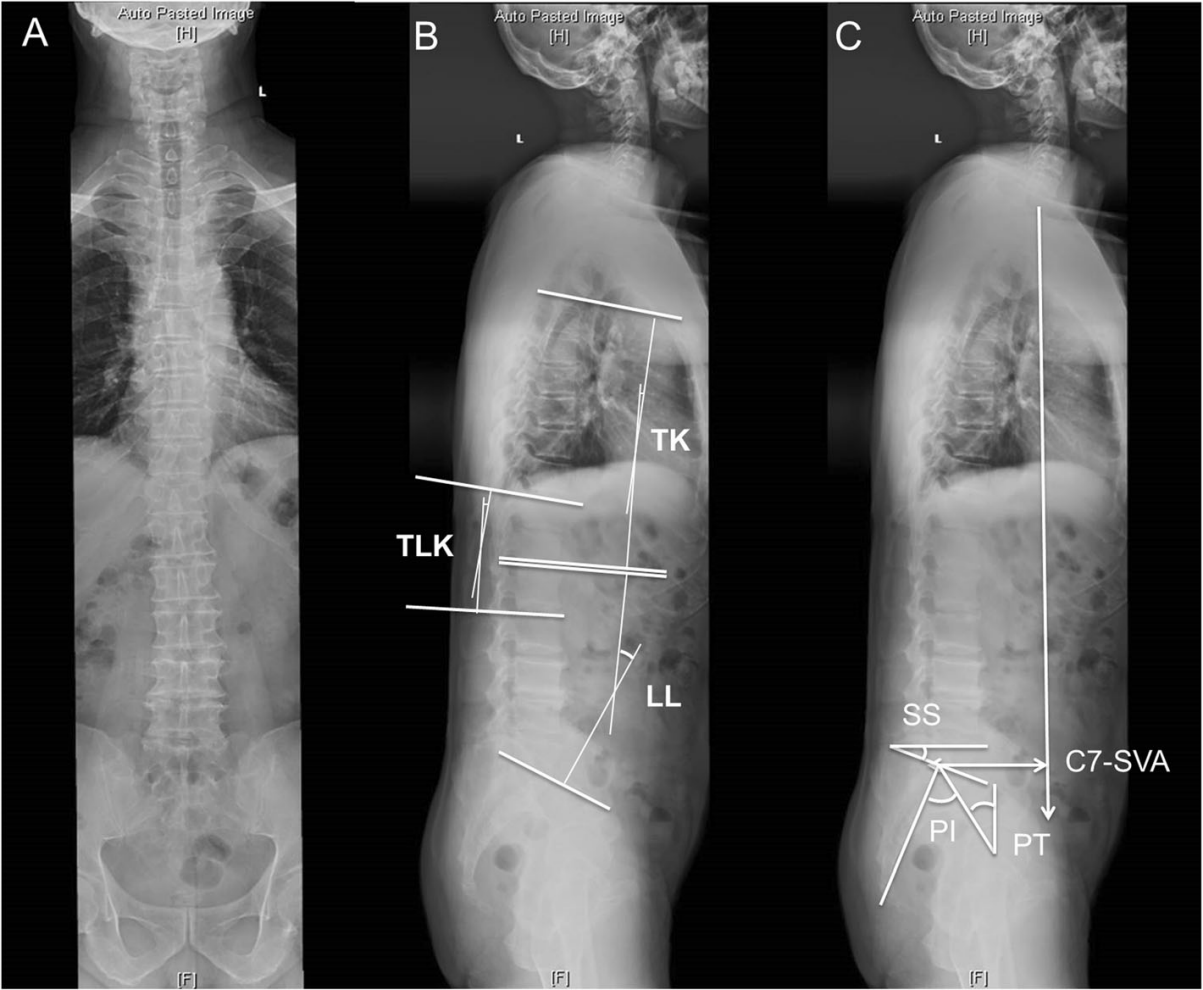
Spinal-pelvic parameters measurements. The spinal-pelvic parameters include: TK: thoracic kyphosis; TLK: Thoracolumbar kyphosis; LL: Lumbar lordosis; PI: Pelvic incidence; SS: Sacral slope; PT: pelvic tilt; C7-SVA: C7 sagittal vertical axis

The same method for measuring spinal-pelvic parameters was used as described in our last study [3]. On the whole spine lateral X-rays, the measurements of the spine parameters include: C7 sagittal vertical axis (C7-SVA); thoracic kyphosis (TK); thoracolumbar kyphosis (TLK); lumbar lordosis (LL). The method of measuring the angle is Cobb method. For TK, TLK, and LL, lordosis was defined positive and kyphosis was defined negative [3]. The measurements of the pelvis parameters include: pelvic incidence (PI); sacral slope (SS); pelvic tilt (PT) [3] (Fig. 2). The DSK patients were divided into four types according to the study of Takemitsu et al. [2, 3].

### Statistical analyses

Statistical Package for Social Science (SPSS, v23.0) software was used for data analysis. The comparison of FI and RCSA for individual muscle between each spinal level used one-way ANOVA with multiple comparisons. The comparison of FI and RCSA among three muscles at each spinal level used one-way ANOVA with multiple comparisons. Bonferroni adjustment was used for multiple comparisons. The correlation between individual muscle degeneration at each lumbar spinal level and spinal-pelvic parameters was analyzed by Pearson correlation test. The data is presented as mean values±SEM (standard error of the mean). *P*-value< 0.05 was considered to be statistically significant.

## Results

### Demographic data

Thirty-two DSK patients (22 females) with age ranging from 48 to 82 years (age = 64.3 ± 8.3 years, mean ± SD) with complete image data were included in this study. According to Takemitsu classification method for degenerative kyphosis, the patients included 11 cases of type I (34%), 15 cases of type II (47%), 6 cases of type III (19%). According to the BMI classification, 3 patients (9%) were in normal weight, 8 patients (25%) were overweight, 21 patients (66%) were obese.

### Back muscle degeneration

**Fig. 3:**
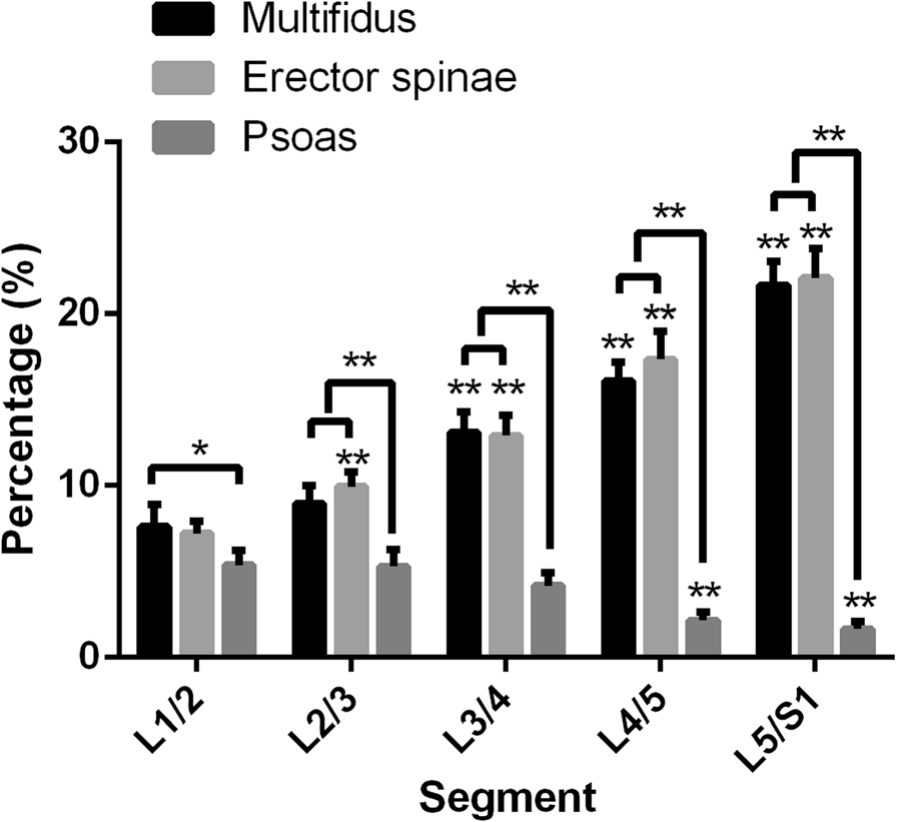
Fat infiltration (FI) of multifidus, erector spinae and psoas at each spinal level from L1/2 to L5/S1. *:< 0.05; **:< 0.01

The fat infiltration (FI) of multifidus at L3/4, L4/5 and L5/S1 were higher than that at L1/2 and L2/3 (*p* < 0.01). The FI of erector spinae at L1/2 was lower than that at all the other spinal levels (*p* < 0.01); the FI at L2/3 was lower than that at L3/4 (*p* < 0.05), L4/5(*p* < 0.01) and L5/S1(*p* < 0.01); the FI at L3/4 was lower than that at L5/S1(*p* < 0.01). The FI of psoas at L1/2, L2/3 and L3/4 were higher than that at L4/5 and L5/S1(*p* < 0.01). At L1/2 spinal level, the FI of multifidus was higher than that of psoas (*p* < 0.05). At L2/3, L3/4, L4/5 and L5/S1spinal level, the FI of multifidus and erector spinae were higher than that of psoas (*p* < 0.01) (Fig. 3).

**Fig. 4:**
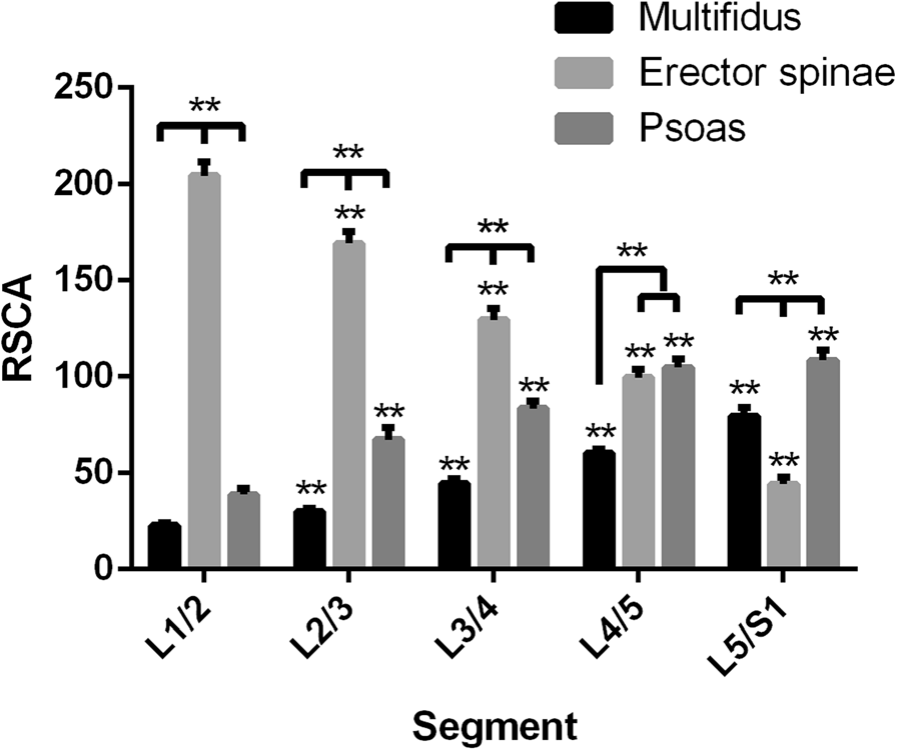
Relative cross-section area (RCSA) of multifidus, erector spinae and psoas at each spinal level from L1/2 to L5/S1. *:< 0.05; **:< 0.01

The relative cross-sectional area (RCSA) of multifidus at upper spinal level was lower than that at lower spinal level (*p* < 0.01). The RCSA of erector spinae at upper spinal level was higher than that at lower spinal level (*p* < 0.01). The RCSA of psoas at L1/2, L2/3 and L3/4 were lower than that at lower spinal levels (*p* < 0.01); the RCSA of psoas at L4/5 was found not different from that at L5/S1(*p* > 0.05). At L1/2, L2/3 and L3/4 spinal level, the RCSA of erector spinae was higher than multifidus and psoas (*p* < 0.01); the RCSA of psoas was higher than multifidus (*p* < 0.01). At L4/5 spinal level, the RCSA of erector spinae and psoas were higher than that of multifidus (*p* < 0.01). At L5/S1 spinal level, the RCSA of psoas was higher than that of multifidus and erector spinae (*p* < 0.01); the RCSA of multifidus was higher than that of erector spinae (*p*< 0.01) (Fig. 4).

### Spinal-pelvic parameters

**Fig. 5:**
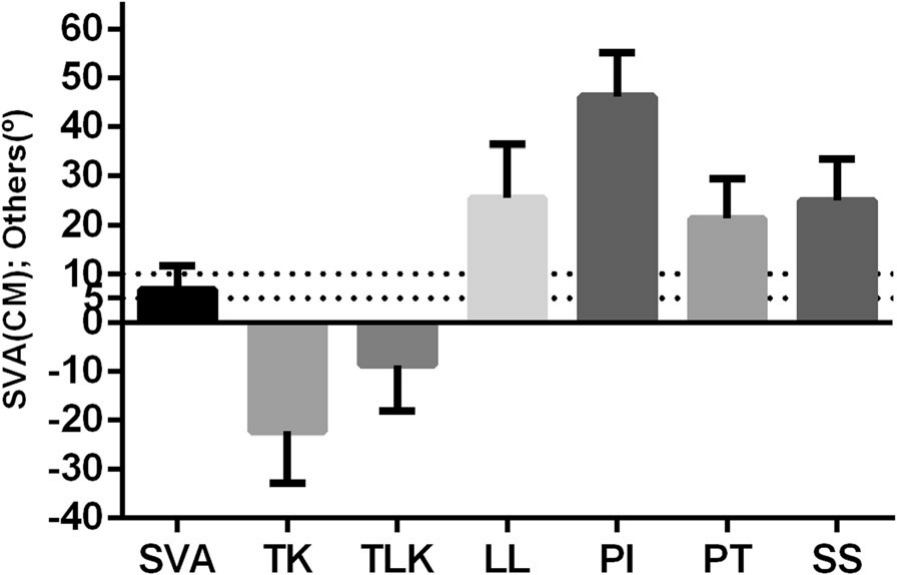
Spinal-pelvic parameters in DSK patients. Mean ± SEM

The angle of spinal kyphosis was defined negative and the angle of spinal lordosis was defined positive. The spinal-pelvic parameters were as follows: SVA value was 6.8 ± 4.8 cm (17 patients (53%) > 5 cm; 21 patients (66%) > 4 cm); TK was − 22.2 ± 10.6°; TLK was − 8.7 ± 9.5°; LL was 25.5 ± 11°; SS was 24.9 ± 8.6°; PT was 21.3 ± 8.1°; PI was 46.2 ± 9.0°(Fig. 5).

### Correlation analysis

**Fig. 6:**
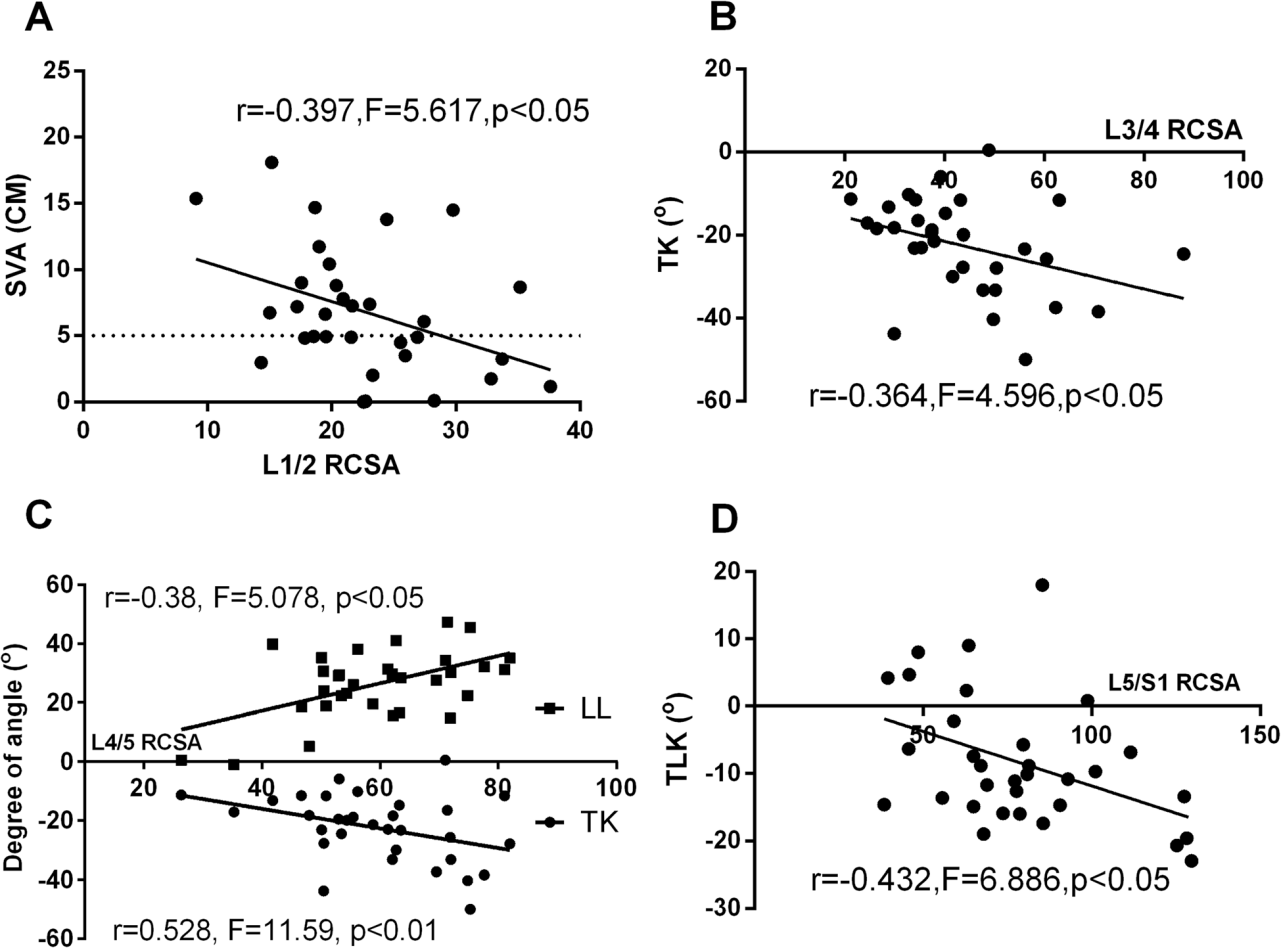
Correlations analysis between RCSA of MF and spinal-pelvic parameters. **a.** RCSA of MF at L1/2 vs. SVA. **b.** RCSA of MF at L3/4 vs. TK. **c.** RCSA of MF at L4/5 vs. LL/TK. **d.** RCSA of MF at L5/S1 vs. TLK

**Fig. 7:**
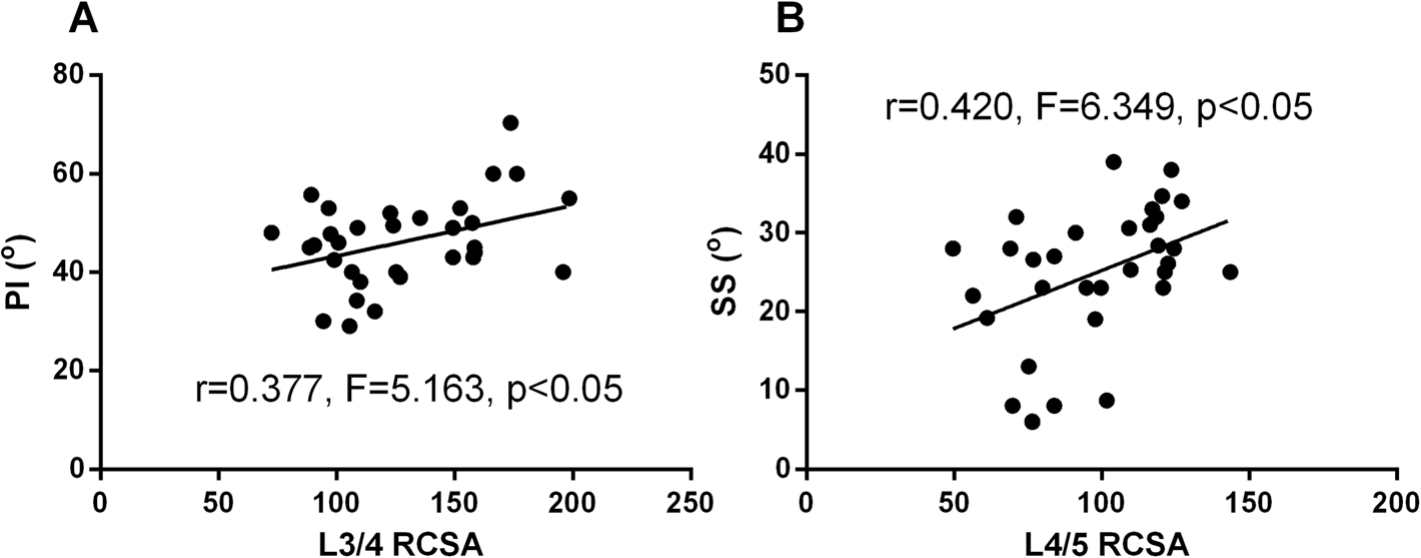
Correlations analysis between RCSA of ES and spinal-pelvic parameters. **a.** RCSA of ES at L3/4 vs. PI. **b.** RCSA of ES at L4/5 vs. SS

In order to determine the influence of individual muscle degeneration at each spinal level on the changes of sagittal spinal alignment, correlation analysis was conducted between the RCSA of multifidus, erector spinae, psoas and the spinal-pelvic parameters. For multifidus, the RCSA at L1/2 spinal level was found negatively correlated with SVA; the RCSA at L3/4 and L4/5 spinal level was found negatively correlated with TK; the RCSA at L4/5 spinal level was found positively correlated with LL; the RCSA at L4/5 and L5/S1 spinal level was found negatively correlated with TLK (Table 1, Fig. 6). For erector spinae, the RCSA at L3/4 spinal level was found positively correlated with PI; the RCSA at L4/5 spinal level was found positively correlated with SS (Table 2, Fig. 7). For psoas, however, the RCSA at any spinal level was not found correlated with the spinal-pelvic parameters (Table 3).

**Table 1:** Correlations between RCSA of multifidus and spinal-pelvic parameters

	SVA	TK	TLK	LL	PI	PT	SS
L1/2 RCSA	**r = − 0.397,** ***p*** = 0.024*	r = − 0.224, *p* = 0.217	r = 0.055, *p* = 0.766	r = 0.006, *p* = 0.973	r = 0.154, *p* = 0.408	r = 0.119, *p* = 0.525	r = 0.049, *p* = 0.792
L2/3 RCSA	r = 0.064, *p* = 0.734	r = 0.089, *p* = 0.628	r = − 0.052, *p* = 0.776	r = 0.222, *p* = 0.223	r = 0.232, *p* = 0.208	r = −0.073, *p* = 0.695	r = 0.313, *p* = 0.087
L3/4 RCSA	r = 0.218, *p* = 0.239	**r = −0.364,*p*** = 0.040*	r = −0.217, *p* = 0.234	r = 0.240, *p* = 0.185	r = 0.178, *p* = 0.338	r = 0.009, *p* = 0.961	r = 0.178, *p* = 0.338
L4/5 RCSA	r = −0.116, *p* = 0.533	**r = −0.380,** ***p*** = 0.032*	**r = − 0.336,** ***p*** = 0.048*	**r = 0.528,*p*** = 0.002*	r = 0.143, *p* = 0.442	r = −0.116, *p* = 0.533	r = 0.261, *p* = 0.157
L5/S1 RCSA	r = − 0.129, *p* = 0.488	r = −0.259, *p* = 0.152	**r = − 0.432,*p*** = 0.014*	r = 0.129, *p* = 0.481	r = − 0.090, *p* = 0.631	r = − 0.005, *p* = 0.980	r = − 0.089, *p* = 0.634

**Table 2:** Correlations between RCSA of erector spinae and spinal-pelvic parameters

	SVA	TK	TLK	LL	PI	PT	SS
L1/2 RCSA	r = 0.030, *p* = 0.873	r = -0.230, *p* = 0.205	r = -0.138, *p* = 0.452	r = 0.082, *p* = 0.654	r = 0.323, *p* = 0.076	r = 0.242, *p* = 0.190	r = 0.111, *p* = 0.552
L2/3 RCSA	r = 0.222, *p* = 0.221	r = −0.101, *p* = 0.581	r = −0.008, *p* = 0.966	r = 0.222, *p* = 0.223	r = 0.319, *p* = 0.081	r = 0.262, *p* = 0.155	r = 0.086, *p* = 0.644
L3/4 RCSA	r = 0.102, *p* = 0.586	r = 0.154, *p* = 0.399	r = −0.183, *p* = 0.317	r = 0.179, *p* = 0.326	**r = 0.377,** ***p*** = 0.037*	r = 0.140, *p* = 0.454	r = 0.263, *p* = 0.153
L4/5 RCSA	r = 0.338, *p* = 0.058	r = 0.001, *p* = 0.995	r = −0.249, *p* = 0.170	r = 0.319, *p* = 0.075	r = 0.288, *p* = 0.116	r = −0.126, *p* = 0.501	**r = 0.420,** ***p*** = 0.019*
L5/S1 SCSA	r = 0.342, *p* = 0.060	r = −0.048, *p* = 0.793	r = −0.148, *p* = 0.419	r = 0.086, *p* = 0.641	r = 0.109, *p* = 0.561	r = −0.067, *p* = 0.719	r = 0.177, *p* = 0.340

**Table 3:** Correlations between RCSA of psoas and spinal-pelvic parameters

	SVA	TK	TLK	LL	PI	PT	SS
L1/2 RCSA	r = −0.227, *p* = 0.220	r = 0.177, *p* = 0.340	r = 0.055, *p* = 0.769	r = 0.152, *p* = 0.415	r = −0.058, *p* = 0.758	r = −0.148, *p* = 0.428	r = 0.080, *p* = 0.670
L2/3 RCSA	r = −0.305, *p* = 0.095	r = −0.000, *p* = 0.998	r = 0.156, *p* = 0.401	r = 0.130, *p* = 0.486	r = −0.151, *p* = 0.418	r = −0.098, *p* = 0.598	r = − 0.065, *p* = 0.729
L3/4 RCSA	r = −0.040, *p* = 0.831	r = 0.199, *p* = 0.282	r = 0.220, *p* = 0.235	r = 0.170, *p* = 0.360	r = −0.018, *p* = 0.924	r = −0.037, *p* = 0.842	r = 0.054, *p* = 0.773
L4/5 RCSA	r = 0.110, *p* = 0.556	r = 0.076, *p* = 0.683	r = 0.141, *p* = 0.449	r = 0.180, *p* = 0.331	r = 0.146, *p* = 0.434	r = 0.033, *p* = 0.861	r = 0.122, *p* = 0.515
L5/S1 RCSA	r = 0.030, *p* = 0.873	r = 0.199, *p* = 0.282	r = 0.310, *p* = 0.090	r = −0.047, *p* = 0.800	r = 0.078, *p* = 0.676	r = 0.167, *p* = 0.369	r = −0.076, *p* = 0.684

## Discussion

### Characteristics of back muscle degeneration in DSK patients

A decrease of muscle size (CSA) and a increase of fatty infiltration (FI) are considered to be two indications of muscle degeneration [14, 16]. Several studies have evaluated the paraspinal muscle composition and morphology in patients with spinal disorders over the last years; they have suggested that both paraspinal muscles CSA and FI are associated with spinal symptoms, including low back pain, radiculopathy, and spinal stenosis [17–19]. The paravertebral back muscles in patients with degenerative flat back showed significant fat infiltration compared with those in the healthy subjects [7]. Previous studies used average FI, CSA or RCSA from L1/2 to L5/S1 for comparing the individual muscle degeneration between groups, however, it is still unclear how they change from L1/2 to L5/S1 for individual muscle and whether they are different between muscles at the same spinal level. In the present study, we used the same grey scale range for every muscle at each spinal level, i.e., 0~120, which allows for comparisons between muscles at specific spinal level and between levels for each muscle [15]. A previous study showed that the FI of multifidus and erector spinae were significantly higher in the degenerative lumbar kyphosis patients than in the healthy volunteers at all levels except L1 [11]. This may be explained by our study that the FI of multifidus and erector spinae increased from L1/2 to L5/S1. Therefore, the significant higher degree of FI was more prevalent to occur at lower lumbar levels compared to upper lumbar levels. The FI of multifidus and erector spinae were also found to be greater at lower spinal level at symptomatic sway-back patients and patients with low back pain [15, 17].

RCSA, which reduces the bias due to relative body size of each individual, might reflect the severity of degenerative spinal disorders [11]. The RCSA of multifidus and psoas increased from L1/2 to L5/S1, whereas the RCSA of erector spinae decreased. The RCSA of erector spinae was higher than multifidus and psoas in upper lumbar spinal levels, whereas, it was the converse in the lower lumbar spinal levels. This indicated that the degeneration of erector spinae is getting worse from L1/2 to L5/S1 relative to multifidus and psoas. The role of erector spinae at lower lumbar spinal level is severely lost. This is consistent with a previous study that the lumbar muscularity of the erector spinae and multifidus were lower in the degenerative lumbar kyphosis patients than in the healthy volunteers at L4 and L5 spinal level [11].

The FI of multifidus and erector spinae were found to be higher than psoas, but the RCSA of psoas becomes more greater especially at lower spinal levels. From our results, we can see that the degeneration of psoas was the lightest except L1/2. This is supported by the study that the FI of psoas in degenerative lumbar kyphosis patients at every spinal level was not different from that in healthy volunteers [11]. The FI in psoas was also found less apparent than in the extensor muscles in flat back patients [7]. The discrepancy in muscle degeneration could be due to:1) The spinal kyphosis overlengthen the extensor muscles which may aggravate the dysfunction and degeneration of extensor muscles but less affect the flexor muscles. 2) There was a apparent effect of lumbar curvature on lever arm lengths for the back extensor muscles, i.e., the lever arm lengths of the erector spinae in lumbar lordosis were significantly longer than in lumbar kyphosis for all spinal levels [20]. This may lead to a decreased movement of lumbar spine in kyphosis patients which may cause the degeneration of the extensor muscle. 3) The spinal sagittal imbalance may cause the discrepancy in muscle degeneration between extensor and flexor muscles [7].

### Correlation between back muscle degeneration and spinal-pelvic parameters in DSK patients

The muscular system plays an essential role in the maintenance of postural balance and the lumbar muscle is important for lumbar segmental stability [18, 21, 22]. Therefore, the defects in the paraspinal muscles are thought to aggravate spinal deformity, i.e., affecting the sagittal and/or coronal balance of the spine column. However, only a limited number of reports have used radiographic methods to assess anatomical changes in the paraspinal muscles of patients with spinal sagittal deformities [7, 11, 15]. Furthermore, until now there were no reports about the influence of individual muscle degeneration at specific spinal level on the sagittal spinal deformity. Therefore, in the present study, we analyzed the correlation between back muscles muscularity (RCSA) and spinal-pelvic parameters in DSK patients. RCSA is considered to be a better indication for evaluating the muscle strength for maintaining the sagittal alignment of the spine [7, 15, 23]. Therefore, it is believed that RCSA may correlate with the level of functional impairment in the back muscle in DSK patients.

The multifidus muscle is located deeply, attaching to the lumbar vertebrae, and is considered responsible for small movements to stabilize the spine and maintain the lumbar curvature [24]. We found that the RCSA of multifidus from L2/3 to L4/5 and RCSA of erector spinae at L4/5 were significantly positively correlated in varying degrees with LL, which indicates that mainly the degeneration of multifidus affects the sagittal spine curvature. In Mitsuru et al.’s study, the L5/S1 multifidus CSA is also found to be significantly correlated with sagittal spinal alignment in degenerative spinal scoliosis patients [10]. Another study showed that the volume of the lumbar extensor muscles in the lower half of the lumbar spine (caudal to the level of the L3/L4 disc) has a positive correlation with the magnitude of the sagittal lumbar curvature over the same region [25]. The force-generating capacity of a muscle is related to its physical size and larger muscle forces would be required to provide stability in lumbar spines that had larger curvatures [26, 27]. The reduction of the strength of the spinal muscles is positively correlated with a reduction of the lumbar curvature. In the present study, the higher RCSA at lower lumbar region is accompanied with larger degrees of LL and TK, this can be explained by the compensation between TK and LL in order to keep the stability of the spine. In addition, we found that the RCSA of multifidus at L1/2 was negatively correlated with SVA. The possible reason could be that in the second layer of multifidus, the distal aspect of muscle fibers that originated from the fascicle from L2 insert into the facet capsule and mamillary process of L5, while those from L3 and more caudal levels insert into the iliac crest, sacroiliac joint, and the sacrum [28]. The muscle strength at L2 is greater than above. Therefore, the multifidus above L2 may mainly maintain the lumbar curvature, but those below L2 may mainly control the rotation of the lumbar spine. Thus, the degeneration of multifidus at this level may aggravate the TLK which may increase SVA and disturb the sagittal balance.

The erector spinae, which is situated more superficially and spans larger sections of the spine, is considered to have a greater role in producing spinal movement [29]. We found that the RCSA of erector spinae at L3/4 was positively correlated with PI and RCSA at L4/5 was positively correlated with SS in DSK patients. This indicates that mainly the degeneration of erector spinae at lower lumbar levels correlate with the changes of pelvic parameters. Pelvic incidence represents a constitutional anatomic parameter in each individual. An increased pelvic incidence is usually associated with a high sacral slope [30]. The erector spinae connects to the posterior pelvis and sacrum which to some extent would control the orientation of the sacrum. The orientation of sacrum can definitely affect the measurements of PI and SS. It was reported that the lower erector spinae obliquity is more pronounced at the level of L4 and L5, and in this region the fascicles of the muscle are capable of generating 40–49% of their total resultant force in the posterior direction [31]. Therefore, the fat infiltration and atrophy of erector spinae at the lower lumbar level would more likely reduce the muscle function which may affect the pelvic parameters.

No significant correlation was found between RCSA of psoas and the spinal-pelvic parameters at any specific spinal level. In Mitsuru et al.’s study, the L5/S1 psoas CSA also has almost no correlation with sagittal spinal alignment in degenerative lumbar scoliosis patients [10]. The psoas muscle is primarily a hip flexor; however, there is some evidence to suggest that it also acts as a spine stabilizer [19, 32]. In the present study, very little fatty infiltration was present in the psoas muscle. It was also reported that the RCSA in low back pain patients with Modic changes in the vertebrae body is bigger than the healthy control people indicating that psoas muscle becomes more active regardless of the presence of degenerative changes of the lumbar spine [23]. Therefore, it could be speculated that in DSK patients, due to 1) the instability and kyphosis of the spine, and 2) DSK patients have more difficulties doing spine extension, the psoas muscle (flexor) may have more movement compared to extensor muscles. However, from the present study, it is still impossible to establish the nature of the causal relationship between spine sagittal deformity and muscle degeneration, i.e., whether the muscle degeneration leads to the DSK or the muscle degeneration is secondary to the DSK.

In addition, spinal and pelvic balance is dependent not only on the paravertebral muscular tension but also on the degeneration and deformity of the spinal column. In the present study, the collapse of the intervertebral disc and the degeneration of the vertebral body also existed, such as Modic changes. The pathology may affect the sagittal balance and have possible additional roles in kyphotic configuration.

### Study limitations

There are some limitations in our study which need further discussion and investigation. First, this study may have been limited by the small number of patients. The scarcity of patients who have degenerative kyphosis deformity and who underwent MRI examination of the lumbar spine was the main causative factor. A large population lased multicenter investigation will be more meaningful and help to clarify and determine the associations between individual back muscle degeneration at specific spinal level and the spinal sagittal alignment. Second, a control group of normal healthy subjects which was lacked in our study would help to observe the muscle degeneration in DSK patients. Third, the fat infiltration of muscles could also be affected by the position of apical vertebrae (e.g., at thoracolumbar or lumbar part). However, due to the lack of type III and IV patients, this question needs further study. Fourth, this is a retrospective, cross-sectional study. Therefore, the causal relationship between the back muscle degeneration and degenerative spinal kyphosis is still unclear. Further studies, such as long-term follow-up studies, will be required to clarify these issues.

## Conclusions

From the present study, we can see that multifidus and erector spinae have different roles in affecting the spinal-pelvic alignment and maintaining the sagittal balance. Multifidus at the lower lumbar spine level is critical for maintaining the curvature of the lumbar spine, whereas erector spinae at the lower lumbar level mainly affects the pelvic parameters. However, psoas seems not to be critically correlated with the changes of spinal-pelvic configuration in DSK patients. The function of the individual muscle at different segments may be different and pay different roles in keep spinal sagittal balance. The present study may provide suggestions trying to avoid iatrogenic injury for the back muscle at specific spinal level during the lumbar spine surgeries. This will help to maintain the sagittal balance post surgeries.

## Data Availability

The datasets used and/or analyzed during the current study are available from the corresponding author on reasonable request.

## Abbreviations

CSA: Cross-sectional area
DSK: Degenerative spinal kyphosis
ES: Erector spinae
FI: Fat infiltration
LL: Lumbar lordosis
MF: Multifidus
MRI: Magnetic resonance imaging
PI: Pelvic incidence
PS: Psoas muscle
PT: Pelvic tilt
RCSA: Relative cross-sectional area
ROI: Region of interest
SS: Sacral slope
SVA: Sagittal vertical axis
TK: Thoracic kyphosis
TLK: Thoracolumbar kyphosis
